# Perceived doctor-patient relationship and satisfaction with general practitioner care in older persons in residential homes

**DOI:** 10.1080/02813432.2018.1459229

**Published:** 2018-04-12

**Authors:** Claudia S. de Waard, Antonius J. Poot, Wendy P. J. den Elzen, Annet W. Wind, Monique A. A. Caljouw, Jacobijn Gussekloo

**Affiliations:** Department of Public Health and Primary Care, Leiden University Medical Center, Leiden, The Netherlands

**Keywords:** Doctor-patient relationship, satisfaction, general practitioner, older persons, residential home, primary care

## Abstract

**Objective:** Understanding patient satisfaction from the perspective of older adults is important to improve quality of their care. Since patient and care variables which can be influenced are of specific interest, this study examines the relation between patient satisfaction and the perceived doctor-patient relationship in older persons and their general practitioners (GPs).

**Design:** Cross-sectional survey.

**Subjects and setting:** Older persons (*n* = 653, median age 87 years; 69.4% female) living in 41 residential homes.

**Main outcome measures:** Patient satisfaction (report mark) and perceived doctor-patient relationship (Leiden Perioperative care Patient Satisfaction questionnaire); relationships were examined by comparing medians and use of regression models.

**Results:** The median satisfaction score was 8 (interquartile range 7.5–9; range 0–10) and doctor-patient relationship 65 (interquartile range 63–65; range 13–65). Higher satisfaction scores were related to higher scores on doctor-patient relationship (Jonckheere Terpstra test, *p* for trend <.001) independent of gender, age, duration of stay in the residential home, functional and clinical characteristics. Adjusted for these characteristics, per additional point for doctor-patient relationship, satisfaction increased with 0.103 points (β = 0.103, 95% CI 0.092–0.114; *p* < .001). In those with a ‘low’ doctor-patient relationship rating, the percentage awarding ‘sufficient or good’ to their GP for ‘understanding about the personal situation’ was 12%, ‘receiving attention as an individual’ 22%, treating the patient kindly 78%, and being polite 94%.

**Conclusion:** In older persons, perceived doctor-patient relationship and patient satisfaction are related, irrespective of patient characteristics. GPs may improve patient satisfaction by focusing more on the affective aspects of the doctor-patient relationship.

**Key Points**
- Examination of the perceived doctor-patient relationship as a variable might better accommodate patients’ expectations and improve satisfaction with the provided primary care.

## Main statements

In older persons, a better perceived doctor-patient relationship relates to higher satisfaction with provided primary care. There is little room for improvement in the formal aspects of the relationship, such as being knowledgeable and polite. However, there is room for improvement in the more affective aspects of the relationship, such as paying attention to the patient’s personal situation and to the patient as an individual.

## Introduction

The widespread use of ‘patient satisfaction’ in the evaluation of care seems justified, considering its importance to all parties concerned. For example, for patients, satisfaction is reported to lead to greater adherence to treatment goals and recommendations [1,2]. For doctors it is relevant that patient satisfaction is positively related to higher staff satisfaction and less malpractice [2], and for policymakers the evaluation of patient satisfaction allows identification of areas for care improvement [1]. For all these parties, it is relevant that patient satisfaction is related to care outcomes and is used as an indicator of quality of care [3,4].

Patient satisfaction can be defined as “*evaluation based on the fulfilment of expectations*” [5]. It is a relative and subjective concept and no simple measure is available to quantify it. Its relation to quality of care is unclear since, for patients, it is difficult to judge the competence of the doctor, and satisfaction implies that an adequate or acceptable standard has been achieved, but not superior service(s) [2–4,6].

Many factors affect patient satisfaction, including the organisational aspects of care and the physical environment. Importantly the characteristics of the patient and doctor influence patient satisfaction [6–8]. From a patient perspective, examples include age, health status, expectations, trust, beliefs, values, and experiences [6,9,10]. Characteristics of doctors which (might) be related to patient satisfaction include age, gender, and attitude. The doctor-patient relationship is important in that it is determined by both parties [6–8,11–14].

Although the above-mentioned factors are related to patient satisfaction, many of them cannot be modified. An exception is the attitude of the doctor as one of the determinants of the doctor-patient relationship. This is important [6–8] and can be modified. To further clarify the multi-dimensional concept of patient satisfaction, the present study investigated the doctor-patient relationship as perceived by the patient, and its relationship with patient satisfaction. In this study, the doctor-patient relationship is seen as the perception of the patient concerning the amount of caring shown by the doctor and the attitude and behaviour of the doctor towards the patient (e.g., respecting patient privacy, being polite) [15]. Assuming that doctors are able to adapt these skills, the doctor-patient relationship might be a factor that can be modified to improve patient satisfaction with care, thereby making health care more responsive to patients’ wants and needs.

## Material and methods

### Study population

Older persons living in residential homes were selected for this study. These older persons have a high complexity of care needs, and are admitted to a residential home because they are unable to sufficiently coordinate their own domestic/medical care. For these persons, the general practitioner (GP) is the most important primary care provider in the Dutch setting, and these persons have often had the same GP for many years. Due to their age and (lack of) mobility they were all visited by their GP in the residential home. The GPs served these patients in the same way as patients living independently in the community.

This study is embedded in the MOVIT project in which regional implementation of integrated care for older persons living in residential homes was the primary goal. The regional project was performed in 41 residential homes in the Netherlands, and was part of the National Program for Elderly Care [16]. Older persons living in a residential home are free to choose one of the regional GPs. The approximately 300 GPs in the region can have patients in one or more residential homes.

For this study, a cross-sectional survey was performed. From October 2010 until December 2012, independent samples of older persons living in their residential home were taken. All residents were invited, except for those residents with dementia in closed psycho-geriatric wards. Residents were informed by letter. Oral consent for interview was obtained by the research nurse after repeating the study information and procedures.

To have a representative sample per residential home, it was planned to include at least 30 residents per residential home, or at least 50% of the residents in homes with fewer than 60 residents. Where necessary, a random selection of residents was made by ranking names of residents alphabetically and inviting the first consecutive uneven numbers followed (if necessary) by consecutive even numbers.

A research nurse interviewed participants by asking the questions and writing down the answers; each interview lasted about 1 h. The questions about care dependency were completed by the nursing staff. Since the present study focused on the doctor-patient relationship, only residents who reported having consulted their GP in the last 12 months were included in the analysis [17].

The study was approved by the Medical Ethics Committee of the Leiden University Medical Center.

### Study parameters

#### Patient satisfaction

General satisfaction with the GP was recorded as a report mark given in response to the question “*Which report mark do you give your GP*?”. A score of 0 indicates totally dissatisfied and 10 indicates completely satisfied.

#### Doctor-patient relationship

The doctor-patient relationship can be seen as the perception of the patient concerning the caring shown by the doctor, and the attitude and behaviour of the doctor towards the patient. The doctor-patient relationship was measured as a domain of the Leiden Perioperative care Patient Satisfaction questionnaire (LPPSq) [15]. This domain consists of 13 questions (see Appendix 1). Participants were asked to score each question on a 5-point Likert scale; total scores range from 13 (worst) to 65 (best).

To group participants by their level of the perceived doctor-patient-relationship, participants were divided into three groups; these groups were based on the total score of the domain of the LPPSq. For the doctor-patient relationship, a score of 13–51 was considered to be ‘low’, a score of 52–64 ‘medium’, and a score of 65 was considered to be an ‘optimal’ perceived relationship.

#### Socio-demographic characteristics

Information was obtained on age, gender, the duration of stay in the residential home, educational level, and income (basic government allowance only, or also a supplementary pension).

#### Number of diseases and ailments

Self-reported chronic diseases and ailments were grouped within the following 19 items: diabetes mellitus, stroke, heart failure, cancer, chronic obstructive pulmonary disease (COPD, asthma), incontinence, urinary tract infections, arthritis, osteoporosis, hip fracture, other fractures, falls, dizziness, prostatism, depression, anxiety, dementia, hearing impairment, and visual impairment.

#### Cognitive function

Cognitive function was measured using the Mini Mental State Examination (MMSE). The questionnaire consists of 11 questions and instructions about orientation, memory, attention, naming, reading and writing. Scores range from 0 (very impaired) to 30 (not impaired) [18].

#### Care dependency

Care dependency was measured by the Care Dependency Scale (CDS), a tool validated for the assessment of the care dependency status of institutionalised patients. Nursing staff were asked to what extent the resident was able to perform 15 basic care needs. These items were measured on a 5-point Likert scale; the total score ranges from 15 (completely care dependent) to 75 (almost independent). The items covered are: eating and drinking, continence, body posture, mobility, day and night pattern, getting (un)dressed, body temperature, hygiene, avoidance of danger, communication, contact with others, sense of rules and values, daily activities, recreational activities and learning ability [19,20].

#### Wellbeing

Wellbeing was measured by a part of the RAND36 questionnaire. Participants were asked to score their feelings (in the last month) on five topics of mental health: (1) being very nervous, (2) feeling calm and peaceful, (3) feeling despondent and sombre, (4) being happy, and (5) feeling so down that nothing could cheer you up.

Participants could choose between six answer categories ranging from ‘always’ to ‘never’. Total scores range from 0–100 with a higher score indicating better wellbeing.

#### Quality of life

The Visual Analogue Scale (VAS) was used to provide an overall estimation of perceived quality of life. The participant marked a point on a line that they felt represented their perception of their current state, ranging from 0–100mm (worst to best imaginable quality of life) [21].

#### Number of contacts with the GP

Participants were asked to categorise the number of contacts with the GP in the last 12 months: 1; 2–4; 5–9; 10 or more visits.

### Statistical analyses

Categorical variables were expressed in percentages and differences between groups analysed with the Chi-square test (linear-by-linear). Continuous variables were expressed as median and interquartile range (IQR) and differences between groups analysed with the Jonckheere Terpstra test.

The relation between the doctor-patient relationship and patient satisfaction was examined using linear regression models. The first model measured the relationship between these two variables. In the second multivariate model, the following were added: gender, age, educational level, income, duration of stay in the residential home, cognitive function, care dependency, psychological wellbeing, quality of life, number of diseases and ailments, and the number of contacts with the GP in the previous 12 months. Only educational level, income, and the number of contacts were categorical variables, all other variables were continuous variables.

A *p-*value <.05 was considered statistically significant. Analyses were conducted with IBM SPSS Statistics for Windows version 20.0.

## Results

Within the MOVIT study, 1,478 residents participated in the interviews. Participants who reported not having seen their GP in the previous 12 months (*n* = 312) and participants who did not complete the questions about satisfaction and doctor-patient relationship (*n* = 513) were excluded. The non-participants did not differ in baseline characteristics from the participants. This resulted in 653 participants available for the present analysis.

### Participants’ characteristics

Table 1 presents the characteristics of the participants. They had a median age of 87 (IQR 83–91) years and were predominantly female (69%). The median duration of stay in the residential home was 2.4 (IQR 1–5) years. Almost half of the participants (48.2%) had an educational level of primary school or less, and 24.2% of the participants had only a basic government allowance as income. More than half of the participants (64.8%) had 1–4 contacts with their GP in the last 12 months.

**Table 1.** Characteristics of the participants (*n* = 653).

	*n*	
Sociodemographic characteristics		
Female	653	453 (69.4%)
Age (years)	653	87 (83–91)
Educational level (primary school or less)	652	315 (48.2%)
Income (basic government allowance only)	640	155 (24.2%)
Duration of stay in residential home (years)	639	2.4 (1–5)
Functional and clinical characteristics		
Cognitive function (MMSE)	651	27 (23–29)
Care dependency (CDS)	644	69 (61–74)
Psychological wellbeing (RAND36/MDS)	622	76 (60–88)
Quality of life: Visual analogue scale (VAS)	628	70 (60–70)
Number of chronic diseases and ailments	653	5 (4–7)
Number of contacts with GP in last 12 months:	653	
1–4 times		423 (64.8%)
5–9 times		135 (20.7%)
≥ 10 times		95 (14.5%)
Perceived doctor-patient relationship (points)	653	65 (63–65)
Patient satisfaction (range 0–10)	653	8.0 (7.5–9.0)

Numerical data: median (interquartile range, IQR), Categorical data: *n* (%)

### Doctor-patient relationship and experienced satisfaction

The median report mark for satisfaction with the GP was 8 (IQR 7.5–9.0). The median score for the doctor-patient relationship was 65 (IQR 63–65). Table 2 shows that 7.6% (*n* = 50) reported a low perceived doctor-patient relationship, 26.0% a medium perceived doctor-patient relationship (*n* = 170), and 66.3% an optimal perceived doctor-patient relationship (*n* = 433).

**Table 2.** Characteristics of the participants (*n* = 653) based on their scores on perceived doctor-patient relationship.

	Perceived doctor-patient relationship*			
	Low (*n* = 50)	Medium (*n* = 170)	Optimal (*n* = 433)	*p*-value**
Patient satisfaction (report mark, 0–10)	6.0 (5.4–7.0)	8.0 (7.0–8.0)	8.0 (8.0–9.0)	<0.001
Sociodemographic characteristics				
Female	40 (80%)	119 (70%)	294 (68%)	0.115
Age (years)	85.0 (81–90)	87.0 (83–90)	87.2 (83–91)	0.153
Educational level (primary school or less)	22 (44%)	79 (47%)	214 (49%)	0.354
Income (basic government allowance only)	11 (22%)	31 (18%)	113 (26%)	0.122
Duration of stay in residential home (years)	2.6 (0.8–4.5)	2.3 (1.1–4.8)	2.5 (1.2–5.1)	0.456
Functional and clinical characteristics				
Cognitive function (MMSE)	27 (24–29)	27 (24–29)	27 (23–29)	0.759
Care dependency (CDS)	67 (59–73)	69 (62–73)	70 (60–74)	0.742
Psychological wellbeing (RAND36/MDS)	60 (42–72)	72 (60–88)	76 (64–88)	<0.001
Quality of life: Visual analogue scale (VAS)	60 (50–70)	70 (60–70)	70 (60–75)	0.002
Number of diseases and ailments	7 (5–8)	6 (4–7)	5 (3–7)	<0.001
Number of contacts with GP in last 12 months:				0.258
1–4 times	39 (78%)	106 (62%)	278 (64%)	
5–9 times	5 (10%)	41 (24%)	89 (21%)	
≥ 10 times	6 (12%)	23 (14%)	66 (15%)	

* Perceived doctor-patient relationship: low level = 13–51 points; medium = 52–64 points; optimal = 65 points.

** Numerical data: median (interquartile range, IQR), Jonckheere Terpstra p for trend test.

Categorical data: *n* (%), Chi-square test, linear-by-linear.

A better doctor-patient relationship (higher score) was associated with more satisfaction experienced by the participants (*p* for trend <.001). Participants with a ‘low’ perceived doctor-patient relationship had a median score for satisfaction of 6 (IQR 5.4–7.0).

Participants with a ‘medium’ perceived doctor-patient relationship had a median score for satisfaction of 8 (IQR 7.0–8.0), and those with an ‘optimal’ score had a median score for satisfaction of 8 (IQR 8.0–9.0) (Table 2). Between the three groups of ratings of doctor-patient relationship, there were no differences in gender, age, educational level, income and/or duration of stay in the residential home. A better perceived doctor-patient relationship was associated with higher scores for wellbeing. In the group with a ‘low’ perceived doctor-patient relationship the median score was 60 (IQR 42–72), in the ‘medium’ group it was 72 (IQR 60–88), and in the ‘optimal’ group it was 76 (IQR 64–88). Participants with a ‘low’ perceived doctor-patient relationship had significantly more self-reported chronic diseases and ailments compared to the ‘medium’ and ‘optimal’ groups.

### Influence of other characteristics

Higher perceived doctor-patient relation was significantly related to higher satisfaction independent of sociodemographic characteristics including gender, age, educational level, income and duration of stay. This relation was also independent of functional characteristics (MMSE, CDS, RAND36 and VAS) and of clinical characteristics (number of diseases and ailments, number of GP contacts) (see Appendix 2).

In linear regression analysis, per additional point extra for the doctor-patient relationship, satisfaction increased with 0.105 points (β = 0.105, 95% CI 0.095–0.115; *p* < .001). In the multivariate model this estimate did not change with adjustment for socio-demographic, functional and clinical characteristics (β = 0.103, 95% CI 0.092–0.114; *p* < .001).

### Items of the doctor-patient relationship

To examine which items of the doctor-patient relationship showed most room for improvement, the 13 individual items of the doctor-patient relationship domain of the LPPSq were analysed. The items ‘being polite’ and ‘being kind’ were the most highly valued (mean scores of 4.93 and 4.91, respectively) (*n* = 653). Because the scores for ‘medium’ and ‘optimal’ groups were so high that improvement was almost impossible, only the group with a ‘low’ rating for the doctor-patient relationship (*n* = 50) was analyzed (Table 3). In this group, the lowest scores were found for ‘Understanding of the GP about the personal situation’ (12% sufficient or good), ‘Attention for you as an individual’ (22% sufficient or good), and ‘Confidence in the GP’ (24% sufficient or good). Even in this group, high percentages for sufficient or good ratings were found for being knowledgeable (50%), taking privacy into account (64%), treating the patient kindly (78%), and being polite (94%).

**Table 3.** Score for the individual items of the perceived doctor-patient relationship, from the 50 participants with a low perceived doctor-patient relationship.

Item on perceived doctor-patient relationship (adapted LPPSq)	Score: sufficient or good (%)
Did the GP show understanding for your personal situation?	12
Did the GP pay attention to you as an individual?	22
Did you have confidence in the GP?	24
Did the GP pay attention to your questions?	28
Did the GP pay attention to your complaints?	28
Had the GP an open attitude?	30
Did you find the GP professional?	38
Did the GP take into account your personal preferences?	40
Was the GP respectful?	44
Did you find the GP knowledgeable?	50
Did the GP take into account your privacy?	64
Were you treated kindly by the GP?	78
Was the GP polite?	94

LPPSq: Leiden Perioperative Patient Satisfaction questionnaire (score 1–5); GP: general practitioner.

## Discussion

In the present study, a better perceived doctor-patient relationship was related to higher patient satisfaction in older persons in a residential home. This relation was independent of gender, age, duration of stay in the residential home, number of diseases, cognitive function, care dependency, quality of life, and number of contacts with the GP. Many participants reported a high satisfaction score and a good doctor-patient relationship.

Analysis of the group with a ‘low’ rating for the doctor-patient relationship shows there is very little room for improvement in the formal aspects of the relationship, such as being knowledgeable and polite. However, affective aspects, such as attention paying attention to the personal situation and to the patient as an individual, do leave room for improvement. These latter aspects have the potential to be modified. This suggests that GPs can have a favorable influence on patient satisfaction by paying attention to these specific aspects; this could also be taken into account in GP training.

### Strengths and limitations

This study has several strengths. We assume that in the perceptions of the patient, there is a degree of overlap between the concepts of satisfaction and doctor-patient relationship. However, satisfaction seems to be the broader concept of the two, being influenced by the doctor-patient relationship rather than the other way around. Although ‘satisfaction’ and ‘doctor-patient relationship’ are difficult concepts, we considered it necessary to explore the relation between these concepts in more depth. A large population of older persons living in residential homes was selected, because this group often has high medical care dependency and often has the same GP for many years. Few studies have explored this topic in this specific population. Asking participants about their experiences over time helps to ensure that the outcomes will be less influenced by a specific consultation or event. In addition, patients’ satisfaction was measured by asking them to rate only one question, without making any assumptions about what we think might determine their satisfaction. Moreover, the use of a multi-component questionnaire to measure the doctor-patient relationship helped to reveal which items were scored as less optimal, enabling to focus on these specific aspects.

A limitation is the loss of the participants (32%) due to incomplete data on the level of satisfaction and on the doctor-patient relationship; possible reasons for this are that some questions may appear rather difficult, together with the length of the total MOVIT questionnaire. However, this latter group of non-participants shows no difference in baseline characteristics from the included participants.

### Comparison with existing literature

Derksen et al. [22] explored the influence of perceived physician empathy on patient satisfaction and several clinical outcomes; the authors state that more evidence is required to affirm the focus on this aspect of care delivery. The importance of the doctor-patient relationship was earlier reported by Jung et al. [8]. Their study showed that patients found the aspects concerning the doctor-patient relationship to be the most important and the best evaluated aspects of care. Also important, but less valued, are the aspects which are more task-oriented, e.g. ‘Getting through to the practice on the phone’, ‘Explaining what to do if you did not get better’ and ‘Referring’; the authors recommend paying extra attention to these latter aspects [8]. Whereas Jung et al. report that there is room for improvement in the task-oriented aspects of care, the present study shows that, especially the affective aspects of the doctor-patient relationship, show room for improvement. However, the task-oriented outcomes of care and affective aspects of the doctor-patient relationship often go hand in hand. This is illustrated by Thygesen et al. [23] who investigated hospital readmission in which an intervention was implemented whereby the GP and the municipal nurse visited older patients after hospital discharge. No effect was found on hospital readmission or subsequent use of primary or secondary healthcare services. However, during home visits, GPs pay special attention to the individual which might benefit other patient outcomes, such as satisfaction. Our study emphasises that older patients indeed appreciate, and expect, this type of attention.

In the present study, the doctor-patient relationship is seen as the perception of the patient concerning the caring shown by the GP, and the attitude and behavior of the GP towards the patient [15]. In other studies, the term ‘physician empathy’ is often used to distinguish between the level of attitude, competency and behaviour [22,24].

### Implications for clinicians and policymakers

The present study shows that, in these older persons with a median age of 87 years and a high complexity of care needs, patient satisfaction is related to the doctor-patient relationship. Persons with a better perceived doctor-relationship were more satisfied with the care delivered by their GP. Especially the affective aspects offer room for improvement and, therefore, also for increased satisfaction in this group of patients. Assuming that physicians are able to influence the doctor-patient relationship by learning/training communicative skills, this could give GPs a tool to better accommodate the expectations of patients and improve satisfaction with the care provided. These skills should focus on the GP asking (at least) about the patient’s perception and enabling patients to address all the problems that they have [25,26].

Therefore, based on these findings, particularly further personalisation of care warrants attention from doctors and policymakers. Future studies should examine whether patient satisfaction measurably improves when doctors improve their skills related to the doctor-patient relationship.

## Appendix 1. Domain of the Leiden perioperative care patient satisfaction questionnaire

The doctor-patient relationship was measured as a domain of the Leiden Perioperative care Patient Satisfaction questionnaire (LPPSq) [15]: this domain consists of the following 13 questions:

**Table:**

– Did the GP take into account your privacy?
– Did you have confidence in the GP?
– Had the GP an open attitude?
– Was the GP respectful?
– Did the GP show understanding for your situation?
– Was the GP polite?
– Did you find the GP professional?
– Did the GP pay attention to your questions?
– Did the GP pay attention to your complaints?
– Did the GP take into account your personal preferences?
– Did you find the GP knowledgeable?
– Did the GP pay attention to you as an individual?
– Were you treated kindly by the GP?

Participants were asked to score each question on a five-point Likert scale: total scores range from 13 (worst) to 65 (best).

GP: general practitioner.

## Appendix 2. Patient satisfaction with general practitioner care, based on perceived doctor-patient relationship.

**Table:**

			Perceived doctor-patient relationship			
			Low (*n =* 50)	Medium (*n =* 170)	Optimal (*n =* 433)	*p-*value^a^
**Sociodemographic characteristics**						
Gender	Male	*n =* 200	6 (5–7)	8 (7–8)	8 (8–9)	<.001^b^
	Female	*n* = 453	6 (6–7)	8 (7–8)	8 (8–9)	<.001^b^
Age (years)	<87	*n* = 322	6 (6–7)	8 (7–8)	8 (8–9)	<.001
	≥87	*n* = 331	6 (5–8)	8 (7–8)	8 (8–9)	<.001
Educational level (low = primary school or less)	Low	*n* = 315	6 (6–7)	8 (7–8)	9 (8–9)	<.001^b^
	High	*n* = 337	6 (5–7)	8 (7–8)	8 (8–9)	<.001^b^
Income (low = basic government allowance only)	Low	*n* = 155	7 (5–8)	8 (7–8)	8 (8–9)	<.001^b^
	High	*n* = 485	6 (6–7)	8 (7–8)	8 (8–9)	<.001^b^
Duration of stay in residential home (years)	<2.4	*n* = 313	6 (6–7)	8 (7–8)	8 (8–9)	<.001
	≥2.4	*n* = 326	6 (5–7)	8 (7–8)	8 (8–9)	<.001
**Functional and clinical characteristics**						
Cognitive function (MMSE)	<26 pts	*n* = 255	6 (6–7)	8 (7–8)	9 (8–9)	<.001
(range 0–30)	≥26 pts	*n* = 396	6 (5–7)	8 (7–8)	8 (8–9)	<.001
Care dependency (CDS)	<69 pts	*n* = 294	7 (5–7)	8 (7–8)	8 (8–9)	<.001
(range 15–75)	≥69 pts	*n* = 350	6 (5–7)	8 (7–8)	8 (8–9)	<.001
Psychological well‐being (RAND36/MDS)	<76 pts	*n* = 301	6 (5–7)	8 (7–8)	8 (8–9)	<.001
(range 0–100)	≥76 pts	*n* = 321	7 (6–7)	8 (7–8)	8 (8–9)	<.001
Quality of life: Visual analogue scale (VAS)	<70 pts	*n* = 301	6 (5–7)	8 (7–8)	8 (8–9)	<.001
(range 0–100)	≥70 pts	*n* = 327	7 (6–8)	8 (7–8)	8 (8–9)	<.001
Number of diseases and ailments	<5	*n* = 253	6 (6–7)	8 (8–8)	8 (8–9)	<.001
	≥5	*n* = 400	6 (5–7)	8 (7–8)	8 (8–9)	<.001
Number of contacts with GP in last 12 months	1–4 times	*n* = 423	6 (5–7)	8 (7–8)	8 (8–9)	<.001^b^
	≥5 times	*n* = 230	7 (7–7)	8 (7–8)	8 (8–9)	<.001^b^

GP: general practitioner; pts: points.

Median patient satisfaction and interquartile range.

a Numerical data: Jonckheere Terpstra p for trend test.

b Categorical data: Chi-square test linear-by-linear.
